# 1,1′-Di-*tert*-butyl-2,2′,3,3′,4,4′,5,5′-octa­ethyl-1,1′-bis­tannole

**DOI:** 10.1107/S1600536811022951

**Published:** 2011-06-18

**Authors:** Takuya Kuwabara, Masaichi Saito

**Affiliations:** a Department of Chemistry, Graduate School of Science and Engineering, Saitama University, Shimo-okubo, Saitama-city, Saitama 338-8570 Japan

## Abstract

The title compound, [Sn_2_(C_4_H_9_)_2_(C_12_H_20_)_2_], has two 1-stannacyclo­penta­diene skeletons related by inversion symmetry located at the mid-point of the Sn—Sn bond [2.7682 (2) Å]. Thus, the asymmetric unit comprises one half-mol­ecule. The planarity of the stannacyclo­penta­diene ring is illustrated by the dihedral angle of 0.3 (1)°, defined by the C_4_ and C—Sn—C planes. To avoid steric repulsion, the two stannole rings are oriented in an *anti* fashion through the Sn—Sn bond. These structural features are similar to those of other bis­tannoles.

## Related literature

For the synthesis and X-ray diffraction analysis of bi(1,1-stannole)s whose carbon atoms of the five-membered rings have phenyl groups, see: Saito *et al.* (2002 ▶, 2005 ▶). For related literature on bi-, oligo- and poly-(1,1-metallole)s, see: Haga *et al.* (2008 ▶); Kanno *et al.* (1998 ▶); Kim & Woo (2002 ▶); Saito & Yoshioka (2005 ▶); Saito *et al.* (2010 ▶); Sohn *et al.* (1999 ▶, 2003 ▶); Yamaguchi & Tamao (1998 ▶); Yamaguchi *et al.* (1997 ▶, 1999 ▶).
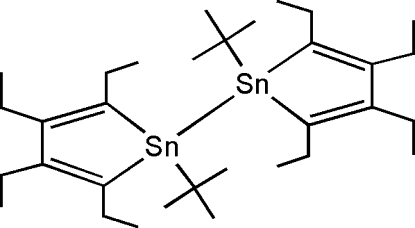


## Experimental

### 

#### Crystal data

[Sn_2_(C_4_H_9_)_2_(C_12_H_20_)_2_]
*M* *_r_* = 680.20
Monoclinic, 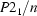

*a* = 8.7161 (5) Å
*b* = 16.5999 (9) Å
*c* = 11.7913 (6) Å
β = 100.827 (1)°
*V* = 1675.67 (16) Å^3^
*Z* = 2
Mo *K*α radiation
μ = 1.51 mm^−1^
*T* = 100 K
0.25 × 0.10 × 0.05 mm

#### Data collection

Bruker APEXII CCD area-detector diffractometer
Absorption correction: multi-scan (*XPREP*; Bruker, 2008 ▶) *T*_min_ = 0.835, *T*_max_ = 0.927
9015 measured reflections
3636 independent reflections
3387 reflections with *I* > 2σ(*I*)
*R*_int_ = 0.017

#### Refinement

*R*[*F* ^2^ > 2σ(*F* ^2^)] = 0.017
*wR*(*F* ^2^) = 0.043
*S* = 1.04
3636 reflections
161 parameters
H-atom parameters constrained
Δρ_max_ = 0.43 e Å^−3^
Δρ_min_ = −0.42 e Å^−3^

### 

Data collection: *APEX2* (Bruker, 2008 ▶); cell refinement: *SAINT* (Bruker, 2008 ▶); data reduction: *SAINT* and *XPREP* (Bruker, 2008 ▶); program(s) used to solve structure: *SHELXS97* (Sheldrick, 2008 ▶); program(s) used to refine structure: *SHELXL97* (Sheldrick, 2008 ▶); molecular graphics: *XSHELL* (Bruker, 2008) ▶; software used to prepare material for publication: *XCIF* (Bruker, 2008) ▶.

## Supplementary Material

Crystal structure: contains datablock(s) I, global. DOI: 10.1107/S1600536811022951/kp2332sup1.cif

Structure factors: contains datablock(s) I. DOI: 10.1107/S1600536811022951/kp2332Isup2.hkl

Supplementary material file. DOI: 10.1107/S1600536811022951/kp2332Isup3.cml

Additional supplementary materials:  crystallographic information; 3D view; checkCIF report

## Figures and Tables

**Table:** 

Sn1—C1	2.1416 (15)
Sn1—C4	2.1475 (16)
Sn1—C5	2.1906 (16)

**Table:** 

C1—Sn1—C4	83.75 (6)

Symmetry code: (i) 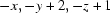.

**Fig. 1.**
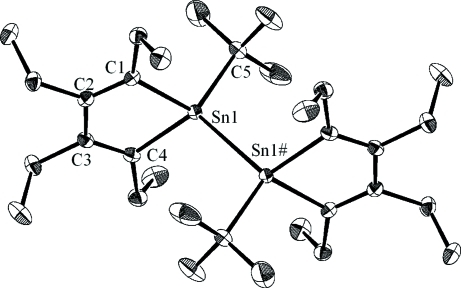
The molecular structure of (I) with atom labels and 50% probability displacement ellipsoids for non-H atoms. H atoms are omitted for clarity. The complete molecule is generated by the symmetry operation: -x, -y+2, -z+1.

**Fig. 2.**
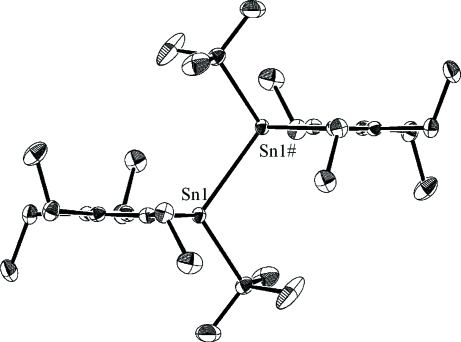
The side view of (I), with atom labels and 50% probability displacement ellipsoids for non-H atoms. H atoms are omitted for clarity.

## supplementary crystallographic information

### Comment

The group 14 metalloles has received much attention as good precursors of their polymers that reveal interesting optical properties (Yamaguchi *et al.*, 1998; Haga *et al.*, 2008) as well as their anion species, which are heavier congeners of the cyclopentadienyl anion (Saito *et al.*, 2005). After the synthesis of several oligo(1,1-silole)s and poly(1,1-silole)s (Yamaguchi *et al.*, 1997, 1999); Kanno *et al.*, 1998; Sohn *et al.*, 1999), they have been used as building blocks of organic electroluminescent devices (Kim & Woo (2002)). Poly(1,1-germole)s have also been synthesized (Sohn *et al.*, 2003). In contrast, as for tin analogues, only a few reports on the synthesis of oligo(1,1-stannole)s have appeared, so far (Haga *et al.*, 2008). We report herein the molecular structure of the title compound, which is a novel bi(1,1-stannole) bearing ethyl groups on the carbon atoms of the five-membered rings.

The X-ray diffraction analysis reveals that the title compound, bis(1-*tert*-butyl-2,3,4,5-tetraethylstannacyclopentadienyl) (I), has two planar five-membered rings with C–C bond alternations. The molecule is centrosymmetric with an inversion center in the middle of Sn–Sn bond, and hence a half moiety of the molecule was refined. The two stannole rings are oriented in an *anti* fashion through the Sn–Sn bond to avoid steric repulsion. The Sn–Sn bond length of 2.7689 (2) Å is in a normal range of the corresponding single bond, as was observed in other bi(1,1-stannole)s (2.7844 (7) and 2.7822 (7) Å (Saito *et al.*, 2002, 2005). The structural features of the title compound are therefore quite similar to those of other bi(1,1-stannole)s that have electronically neutral tin centers, and substituents on the ring carbon atoms little affect the structural feartures of bi(1,1-stannole)s.

### Experimental

A diethyl ether solution (0.55 mL) of *tert*-butyl chloride (0.94 *M*, 0.52 mmol) was added to a diethyl ether solution (7 mL) of 2,2',3,3',4,4',5,5'-octaethyl-1,1'-dilithiobistannole (Saito *et al.*, 2010) (118.8 mg, 0.203 mmol) at room temperature, and the mixture was stirred for 3 h. After removal of volatile substances, the residue was degassed by freeze-pump-thaw cycles and sealed. In a glovebox, materials insoluble in hexane were removed by filtration and the filtrate was concentrated to provide a crude product. Recrystallisation of the crude product from diethyl ether afforded colourless crystals of bis(1-*tert*-butyl-2,3,4,5-tetraethylstannacyclopentadienyl) (107.7 mg, 0.154 mmol, 76%). (1) ^1^H NMR (C_6_D_6_, 400 MHz) δ 1.02 (t, *J* = 7 Hz, 12H), 1.19 (t, *J* = 7 Hz, 12H), 1.40(s, *J*_Sn–H_ = 73 Hz, 18H), 2.33(q, *J* = 7 Hz, 8H), 2.41–2.60(m, 8H); ^13^C NMR (101 MHz, *C*_6_D_6_) δ 18.44 (q, *J*_Sn–C_ = 13 Hz), 22.61 (t, *J*_Sn–C_ = 48 Hz), 26.85 (t, *J*_Sn–C_ = 54 Hz), 30.71 (s, *J*_Sn–C_ = 22, 314, 328 Hz), 32.54 (*t*), 145.74 (s, *J*_Sn–C_ = 26, 287, 301 Hz), 152.92 (s, *J*_Sn–C_ = 21, 63 Hz); ^119^Sn NMR (186 MHz, C_6_D_6_)δ -68.8 (*J*_Sn–C_ = 301 Hz, *J*_Sn–Sn_ = 948 Hz).

### Refinement

All H atoms were positionated geometrically, with C–H 0.96 and 0.97 A for methyl and methylene H atoms, and constrated to ride on their parent atoms, with *U*_iso_(H) = 1.5*U*_eq_(C) and 1.2*U*_eq_(C) for methyl and methylene H atoms, respectively.

### Crystal data

**Table:**

[Sn_2_(C_4_H_9_)_2_(C_12_H_20_)_2_]	*F*(000) = 700
*M**_r_* = 680.20	*D*_x_ = 1.348 Mg m^−^^3^
Monoclinic, *P*2_1_/*n*	Mo *K*α radiation, λ = 0.71073 Å
Hall symbol: -P 2yn	Cell parameters from 6469 reflections
*a* = 8.7161 (5) Å	θ = 2.5–28.1°
*b* = 16.5999 (9) Å	µ = 1.51 mm^−^^1^
*c* = 11.7913 (6) Å	*T* = 100 K
β = 100.827 (1)°	Cube, colourless
*V* = 1675.67 (16) Å^3^	0.25 × 0.10 × 0.05 mm
*Z* = 2	

### Data collection

**Table:**

Bruker APEXII CCD area-detector diffractometer	3636 independent reflections
Radiation source: Bruker TXS fine-focus rotating anode	3387 reflections with *I* > 2σ(*I*)
Bruker Helios multilayer confocal mirror	*R*_int_ = 0.017
Detector resolution: 8.333 pixels mm^-1^	θ_max_ = 27.0°, θ_min_ = 2.1°
φ and ω scans	*h* = −11→9
Absorption correction: multi-scan (*XPREP*; Bruker, 2008)	*k* = −21→21
*T*_min_ = 0.835, *T*_max_ = 0.927	*l* = −14→12
9015 measured reflections	

### Refinement

**Table:**

Refinement on *F*^2^	Primary atom site location: structure-invariant direct methods
Least-squares matrix: full	Secondary atom site location: difference Fourier map
*R*[*F*^2^ > 2σ(*F*^2^)] = 0.017	Hydrogen site location: inferred from neighbouring sites
*wR*(*F*^2^) = 0.043	H-atom parameters constrained
*S* = 1.04	*w* = 1/[σ^2^(*F*_o_^2^) + (0.020*P*)^2^ + 0.7365*P*] where *P* = (*F*_o_^2^ + 2*F*_c_^2^)/3
3636 reflections	(Δ/σ)_max_ = 0.002
161 parameters	Δρ_max_ = 0.43 e Å^−^^3^
0 restraints	Δρ_min_ = −0.42 e Å^−^^3^

### Special details

**Table:**

Experimental. (*SADABS*; Bruker, 2008)
Geometry. All e.s.d.'s (except the e.s.d. in the dihedral angle between two l.s. planes) are estimated using the full covariance matrix. The cell e.s.d.'s are taken into account individually in the estimation of e.s.d.'s in distances, angles and torsion angles; correlations between e.s.d.'s in cell parameters are only used when they are defined by crystal symmetry. An approximate (isotropic) treatment of cell e.s.d.'s is used for estimating e.s.d.'s involving l.s. planes.
Refinement. Refinement of *F*^2^ against ALL reflections. The weighted *R*-factor *wR* and goodness of fit *S* are based on *F*^2^, conventional *R*-factors *R* are based on *F*, with *F* set to zero for negative *F*^2^. The threshold expression of *F*^2^ > σ(*F*^2^) is used only for calculating *R*-factors(gt) *etc*. and is not relevant to the choice of reflections for refinement. *R*-factors based on *F*^2^ are statistically about twice as large as those based on *F*, and *R*- factors based on ALL data will be even larger.

### Fractional atomic coordinates and isotropic or equivalent isotropic displacement parameters (Å^2^)

**Table:**

	*x*	*y*	*z*	*U*_iso_*/*U*_eq_	
Sn1	0.025172 (11)	0.980800 (6)	0.390739 (8)	0.01472 (4)	
C1	0.08418 (18)	1.07858 (9)	0.28847 (13)	0.0166 (3)	
C2	−0.03317 (18)	1.08829 (9)	0.19728 (13)	0.0165 (3)	
C3	−0.17491 (18)	1.03462 (9)	0.18331 (13)	0.0166 (3)	
C4	−0.18366 (18)	0.97554 (9)	0.26111 (14)	0.0174 (3)	
C5	0.20071 (18)	0.88519 (10)	0.40268 (14)	0.0207 (3)	
C6	0.2344 (3)	0.86861 (15)	0.28284 (18)	0.0494 (6)	
H6A	0.2631	0.9179	0.2498	0.074*	
H6B	0.1428	0.8468	0.2347	0.074*	
H6C	0.3187	0.8307	0.2884	0.074*	
C7	0.3492 (2)	0.91665 (13)	0.47898 (19)	0.0412 (5)	
H7A	0.4319	0.8783	0.4801	0.062*	
H7B	0.3311	0.9245	0.5560	0.062*	
H7C	0.3781	0.9670	0.4489	0.062*	
C8	0.1474 (3)	0.80959 (13)	0.4556 (3)	0.0577 (7)	
H8A	0.0592	0.7872	0.4042	0.087*	
H8B	0.1182	0.8226	0.5280	0.087*	
H8C	0.2310	0.7710	0.4681	0.087*	
C9	0.23347 (19)	1.12690 (10)	0.31206 (14)	0.0207 (3)	
H9A	0.2443	1.1562	0.2428	0.025*	
H9B	0.3215	1.0904	0.3308	0.025*	
C10	0.2376 (2)	1.18665 (11)	0.41124 (15)	0.0285 (4)	
H10A	0.1539	1.2247	0.3915	0.043*	
H10B	0.3356	1.2147	0.4246	0.043*	
H10C	0.2260	1.1581	0.4799	0.043*	
C11	−0.02473 (19)	1.15157 (10)	0.10545 (13)	0.0213 (3)	
H11A	0.0349	1.1974	0.1409	0.026*	
H11B	−0.1294	1.1701	0.0735	0.026*	
C12	0.0514 (2)	1.11894 (11)	0.00763 (14)	0.0276 (4)	
H12A	0.1557	1.1013	0.0387	0.041*	
H12B	0.0547	1.1607	−0.0482	0.041*	
H12C	−0.0086	1.0744	−0.0289	0.041*	
C13	−0.30873 (19)	1.05102 (10)	0.08346 (14)	0.0229 (3)	
H13A	−0.3647	1.0012	0.0615	0.027*	
H13B	−0.2668	1.0698	0.0176	0.027*	
C14	−0.4225 (2)	1.11386 (11)	0.11409 (18)	0.0348 (4)	
H14A	−0.4702	1.0938	0.1755	0.052*	
H14B	−0.5018	1.1246	0.0475	0.052*	
H14C	−0.3669	1.1627	0.1385	0.052*	
C15	−0.32148 (19)	0.92105 (10)	0.26192 (15)	0.0230 (3)	
H15A	−0.4082	0.9395	0.2037	0.028*	
H15B	−0.3527	0.9253	0.3364	0.028*	
C16	−0.2897 (2)	0.83329 (11)	0.23913 (18)	0.0337 (4)	
H16A	−0.2663	0.8279	0.1631	0.051*	
H16B	−0.3802	0.8017	0.2446	0.051*	
H16C	−0.2024	0.8147	0.2952	0.051*	

### Atomic displacement parameters (Å^2^)

**Table:**

	*U*^11^	*U*^22^	*U*^33^	*U*^12^	*U*^13^	*U*^23^
Sn1	0.01341 (6)	0.01614 (6)	0.01448 (6)	0.00001 (4)	0.00225 (4)	0.00191 (4)
C1	0.0176 (7)	0.0170 (7)	0.0159 (7)	−0.0015 (6)	0.0052 (6)	−0.0001 (6)
C2	0.0193 (7)	0.0139 (7)	0.0172 (7)	0.0008 (6)	0.0058 (6)	−0.0002 (6)
C3	0.0149 (7)	0.0168 (7)	0.0175 (7)	0.0016 (6)	0.0014 (6)	−0.0027 (6)
C4	0.0141 (7)	0.0185 (7)	0.0194 (8)	−0.0002 (6)	0.0028 (6)	−0.0020 (6)
C5	0.0170 (8)	0.0210 (8)	0.0232 (8)	0.0027 (6)	0.0013 (6)	0.0000 (6)
C6	0.0559 (14)	0.0607 (15)	0.0314 (11)	0.0330 (12)	0.0074 (10)	−0.0069 (10)
C7	0.0230 (10)	0.0449 (12)	0.0498 (12)	0.0099 (8)	−0.0082 (8)	−0.0115 (10)
C8	0.0319 (12)	0.0326 (11)	0.112 (2)	0.0118 (9)	0.0221 (13)	0.0330 (13)
C9	0.0193 (8)	0.0216 (8)	0.0210 (8)	−0.0044 (6)	0.0036 (6)	0.0011 (6)
C10	0.0302 (10)	0.0268 (9)	0.0270 (9)	−0.0092 (7)	0.0015 (7)	−0.0042 (7)
C11	0.0240 (8)	0.0190 (8)	0.0203 (8)	−0.0009 (6)	0.0027 (6)	0.0041 (6)
C12	0.0343 (10)	0.0299 (9)	0.0195 (8)	−0.0047 (7)	0.0075 (7)	0.0035 (7)
C13	0.0210 (8)	0.0226 (8)	0.0222 (8)	−0.0001 (7)	−0.0030 (6)	0.0014 (7)
C14	0.0234 (9)	0.0293 (9)	0.0472 (12)	0.0069 (7)	−0.0054 (8)	−0.0003 (8)
C15	0.0178 (8)	0.0241 (8)	0.0268 (8)	−0.0037 (6)	0.0033 (6)	0.0017 (7)
C16	0.0355 (10)	0.0252 (9)	0.0433 (11)	−0.0132 (8)	0.0146 (9)	−0.0065 (8)

### Geometric parameters (Å, °)

**Table:**

Sn1—C1	2.1416 (15)	C9—H9A	0.9700
Sn1—C4	2.1475 (16)	C9—H9B	0.9700
Sn1—C5	2.1906 (16)	C10—H10A	0.9600
Sn1—Sn1^i^	2.7682 (2)	C10—H10B	0.9600
C1—C2	1.347 (2)	C10—H10C	0.9600
C1—C9	1.509 (2)	C11—C12	1.534 (2)
C2—C3	1.507 (2)	C11—H11A	0.9700
C2—C11	1.520 (2)	C11—H11B	0.9700
C3—C4	1.355 (2)	C12—H12A	0.9600
C3—C13	1.518 (2)	C12—H12B	0.9600
C4—C15	1.505 (2)	C12—H12C	0.9600
C5—C8	1.513 (3)	C13—C14	1.529 (2)
C5—C6	1.521 (3)	C13—H13A	0.9700
C5—C7	1.523 (2)	C13—H13B	0.9700
C6—H6A	0.9600	C14—H14A	0.9600
C6—H6B	0.9600	C14—H14B	0.9600
C6—H6C	0.9600	C14—H14C	0.9600
C7—H7A	0.9600	C15—C16	1.516 (2)
C7—H7B	0.9600	C15—H15A	0.9700
C7—H7C	0.9600	C15—H15B	0.9700
C8—H8A	0.9600	C16—H16A	0.9600
C8—H8B	0.9600	C16—H16B	0.9600
C8—H8C	0.9600	C16—H16C	0.9600
C9—C10	1.529 (2)		
			
C1—Sn1—C4	83.75 (6)	C1—C9—H9B	109.1
C1—Sn1—C5	110.28 (6)	C10—C9—H9B	109.1
C4—Sn1—C5	120.31 (6)	H9A—C9—H9B	107.9
C1—Sn1—Sn1^i^	116.51 (4)	C9—C10—H10A	109.5
C4—Sn1—Sn1^i^	114.24 (4)	C9—C10—H10B	109.5
C5—Sn1—Sn1^i^	109.75 (4)	H10A—C10—H10B	109.5
C2—C1—C9	125.63 (14)	C9—C10—H10C	109.5
C2—C1—Sn1	108.27 (11)	H10A—C10—H10C	109.5
C9—C1—Sn1	126.08 (11)	H10B—C10—H10C	109.5
C1—C2—C3	120.03 (13)	C2—C11—C12	112.17 (14)
C1—C2—C11	121.33 (14)	C2—C11—H11A	109.2
C3—C2—C11	118.62 (13)	C12—C11—H11A	109.2
C4—C3—C2	120.22 (14)	C2—C11—H11B	109.2
C4—C3—C13	121.47 (14)	C12—C11—H11B	109.2
C2—C3—C13	118.26 (13)	H11A—C11—H11B	107.9
C3—C4—C15	125.86 (15)	C11—C12—H12A	109.5
C3—C4—Sn1	107.73 (11)	C11—C12—H12B	109.5
C15—C4—Sn1	126.20 (11)	H12A—C12—H12B	109.5
C8—C5—C6	111.14 (18)	C11—C12—H12C	109.5
C8—C5—C7	109.49 (17)	H12A—C12—H12C	109.5
C6—C5—C7	108.65 (17)	H12B—C12—H12C	109.5
C8—C5—Sn1	111.26 (12)	C3—C13—C14	112.16 (14)
C6—C5—Sn1	109.01 (12)	C3—C13—H13A	109.2
C7—C5—Sn1	107.17 (11)	C14—C13—H13A	109.2
C5—C6—H6A	109.5	C3—C13—H13B	109.2
C5—C6—H6B	109.5	C14—C13—H13B	109.2
H6A—C6—H6B	109.5	H13A—C13—H13B	107.9
C5—C6—H6C	109.5	C13—C14—H14A	109.5
H6A—C6—H6C	109.5	C13—C14—H14B	109.5
H6B—C6—H6C	109.5	H14A—C14—H14B	109.5
C5—C7—H7A	109.5	C13—C14—H14C	109.5
C5—C7—H7B	109.5	H14A—C14—H14C	109.5
H7A—C7—H7B	109.5	H14B—C14—H14C	109.5
C5—C7—H7C	109.5	C4—C15—C16	113.80 (14)
H7A—C7—H7C	109.5	C4—C15—H15A	108.8
H7B—C7—H7C	109.5	C16—C15—H15A	108.8
C5—C8—H8A	109.5	C4—C15—H15B	108.8
C5—C8—H8B	109.5	C16—C15—H15B	108.8
H8A—C8—H8B	109.5	H15A—C15—H15B	107.7
C5—C8—H8C	109.5	C15—C16—H16A	109.5
H8A—C8—H8C	109.5	C15—C16—H16B	109.5
H8B—C8—H8C	109.5	H16A—C16—H16B	109.5
C1—C9—C10	112.43 (13)	C15—C16—H16C	109.5
C1—C9—H9A	109.1	H16A—C16—H16C	109.5
C10—C9—H9A	109.1	H16B—C16—H16C	109.5

Symmetry codes: (i) −*x*, −*y*+2, −*z*+1.
